# Heart Transplantation and Donation After Circulatory Death in Children. A Review of the Technological, Logistical and Ethical Framework

**DOI:** 10.3389/ti.2025.13801

**Published:** 2025-02-14

**Authors:** Louise Amelia Kenny, Liz Armstrong, Marius Berman, Joe Brierley, David Crossland, John Dark, Dale Gardiner, Stephen Ralph Large, Derek Manas, Mohamed Nassar, David Shaw, Emma Simpson

**Affiliations:** ^1^ Paediatric Heart Unit, Institute of Transplantation, Freeman Hospital, Newcastle upon Tyne, United Kingdom; ^2^ Congenital Heart Disease Research Group, Population Health Sciences Institute, Newcastle University, Newcastle upon Tyne, United Kingdom; ^3^ National Health Service Blood and Transplant, Bristol, United Kingdom; ^4^ Department of Cardiothoracic Surgery, Papworth Hospital NHS Foundation Trust, Cambridge, United Kingdom; ^5^ Paediatric Intensive Care Unit, Great Ormond Street Hospital for Children NHS Foundation Trust, London, United Kingdom; ^6^ Translational and Clinical Research Institute, Faculty of Medical Sciences, Newcastle University, Newcastle upon Tyne, England, United Kingdom; ^7^ Intensive Care Unit, Nottingham University Hospitals NHS Trust, Nottingham, United Kingdom; ^8^ Faculty of Medicine, Alexandria, Egypt; ^9^ Institute of Biomedical Ethics, University of Basel, Basel, Switzerland; ^10^ Institute of Care and Public Health Research, Faculty of Health, Medicine and Life Sciences, Maastricht University, Maastricht, Netherlands

**Keywords:** pediatric organ donation, pediatric heart transplantation, donation after circulatory death (DCD), hypothermic organ perfusion, *ex-situ* heart perfusion

## Abstract

Heart transplant for adults following Donation after Circulatory Death (DCD) is well established in many parts of the world, including the United Kingdom (UK). Small child DCD hearts have now been recovered in the UK and internationally utilising novel technologies. Despite these recent advances, extension of this practice to pediatric cardiac transplantation has been slow and difficult despite the severe shortage of donors for children leading to a high number of deaths annually of children waiting for heart transplant. This is in direct contrast with the thriving UK programme of adult DCD heart transplant and pediatric DCD donation for non-cardiac organs. There has been insufficient action in addressing this inequality thus far. Barriers to development of a pediatric cardiac DCD programme are multifaceted: ethical concerns, technological paucity, financial and logistical hurdles. We describe the background, live issues, current developments and how we are driving resources toward a sustainable DCD programme for small children in the UK to provide valuable insights to other countries of the elements and principles at play. This is a call to responsible bodies to take urgent and achievable actions to establish an equitable paediatric DCD cardiac programme for donors, recipients and their families.

## Introduction

**FIGURE 1:**
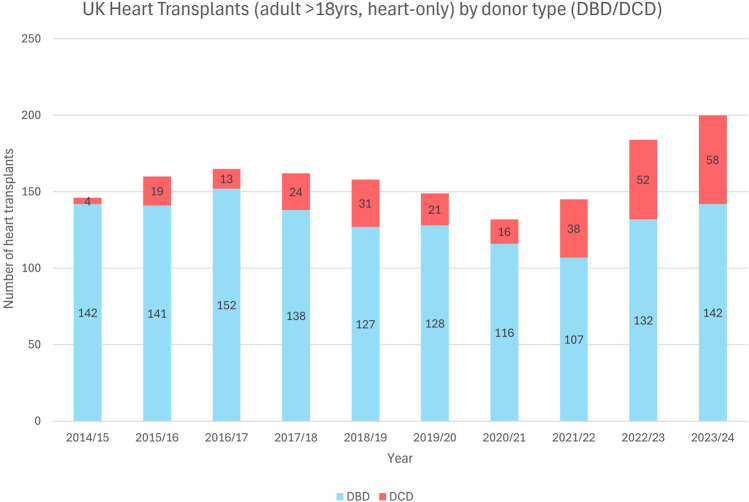
UK heart transplantations (adult >18 years, heart alone) by donor type (DBD/DCD) demonstrating the increasing DCD heart utilization in adults over the past decade (2014–2024).

Controlled donation after circulatory death (DCD) is a well-established practice in the United Kingdom (UK), now accounting for 46% of all deceased donor organs. Since the year 2000, the UK has carried out over 8000 DCD donations providing for over 20,000 recipients [1]. In 2015, the UK was one of the first nations to commence cardiac DCD transplantation and has performed almost 300 heart transplants from DCD donors (see Figure 1) with recipient outcomes comparable to those following Donation after Brain Death (DBD) transplantation [2–4]. Last year, 29% of UK adult heart transplants were made possible by DCD donation and this has given rise to a year-on-year increase in the total number of heart transplants performed [1].

So, what of children? Since commencing in 2013, a total of 200 children (<18 years) in the UK have become DCD donors contributing at least 1 transplantable organ, accounting for almost 40% of UK pediatric donations [1]. However, Paediatric DCD heart donation and transplantation remains a rare event. Only 28 of the 297 UK DCD heart transplants have occurred in recipients <18 years, exclusively in older children and adolescents. Meanwhile, each year, 10–15 children die waiting for a heart in the UK [1]. The DCD pediatric donor pool, accessible for children of any size awaiting liver and kidney, remains inaccessible to small children in need of a heart. Waitlist mortality remains excessively high, in part due to current barriers to smaller DCD heart donors.

We seek here to examine the present technological, logistical and ethical obstacles to achieving a functional cardiac DCD program in children and provide a synopsis of the ethical, clinical and legal framework that already exists to provide the solution to these obstacles. We hope to encourage progress in our own country and provide valuable insight to others considering a cardiac DCD pediatric program.

## Paediatric Cardiac Donation

**FIGURE 2:**
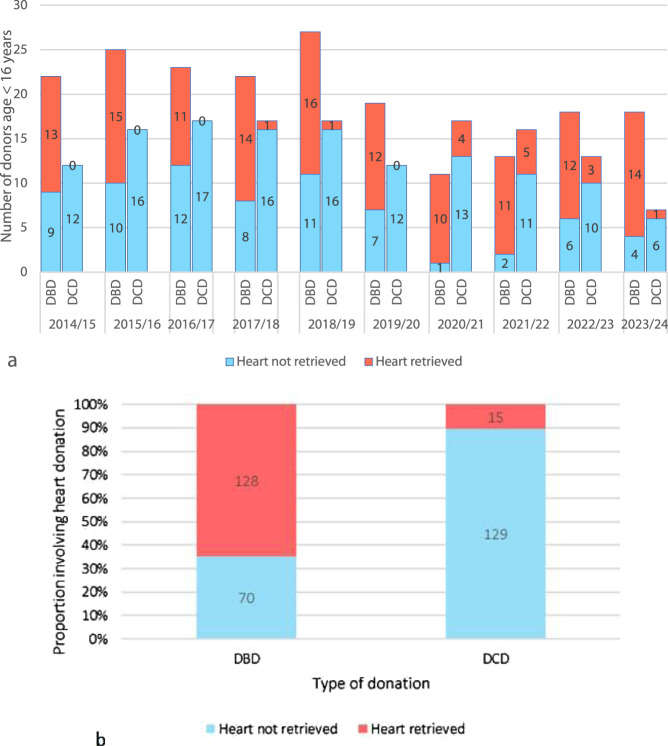
**(A)** An annual breakdown of the past decade of UK pediatric (aged <16 years) solid organ donors by donor type (DBD/DCD) and categorized into heart retrieved and heart not retrieved. **(B)** Total number of DBD and DCD pediatric donors (<16 years) in the past 10 years (2014–2024) categorized into heart retrieved and heart not retrieved) [1].

Over the past decade, although paediatric DBD donors have reduced in numbers overall, the proportion of DBD hearts retrieved has increased (Figure 2A). The majority of paediatric DBD donations include cardiac, demonstrating a willingness from donor families to donate the heart. Conversely, paediatric DCD organ donations rarely include the heart, and numbers have remained low since the introduction of the paediatric DCD cardiac retrieval in 2017 (Figures 2A, B) [1].

**FIGURE 3:**
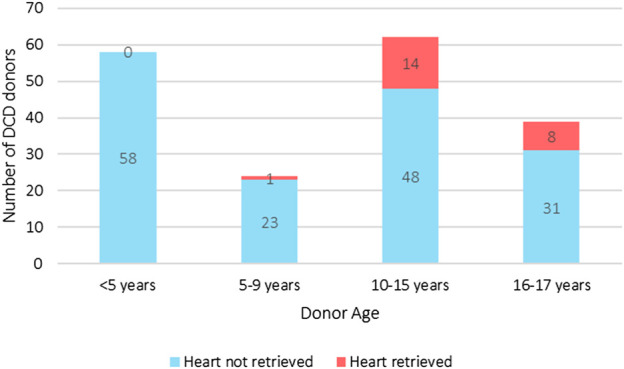
UK pediatric DCD all solid organ donation with and without heart retrieval, and heart donation categorized by age groups (2014–2024). The median weight of the heart donors was 60 kg (IQR 50–70 kg) [1].

As yet, pediatric DCD cardiac donation remains an uncommon occurrence with only fifteen children <16 years old donating DCD hearts (Figure 3) [1]. These children were predominantly adolescents with a median donor weight of 60 kg (IQR 50–70 kg). The leading restriction is that the *ex-situ* normothermic preservation technology used in the UK–the Organ Care System (TransMedics OCS™) – only permits DCD heart retrieval from donors >50 kg which excludes most children from DCD heart donation. The practice of size mismatching enables a 20 kg child to receive a heart from a 50 kg DCD donor, but smaller children are acutely disadvantaged by the donor weight criteria.

Adult data shows that enabling DCD cardiac donation can add significantly to the organ pool (Figure 1). Figures 2A, B suggest there have been a significant number of missed opportunities for heart donation from DCD donors, particularly in the younger age categories.

It is not possible to determine the number of true potential heart donors from this retrospective cohort. Historically, DCD cardiac donation has not been explored in children <50 kg due to lack of technology to retrieve the heart. As such many potential donors did not undergo echocardiography to determine organ suitability, nor were families approached for consent for heart recovery. One could assume that since families consented to donation of other organs, then a number of these DCD donors, represented by blue on Figures 2A, B, may have fulfilled criteria of consent, organ condition and ischemic time. A potential cardiac donor represents a missed opportunity for both donor and recipient patients and their families.

## What is the Clinical Need?

At any given time, there are 40–50 children waiting for a heart-alone transplant in the two national centres across the UK (Freeman Hospital, Newcastle; Great Ormond Street Hospital London – FRH/GOSH). 40% of these children are below 25 kg and therefore unsuitable for DCD hearts utilising TransMedics OCS™.

Many of these children are supported mechanically by ventricular assist devices which require the smaller child to remain an inpatient whilst waiting for an organ. Children on these devices are vulnerable to death, stroke, infection, organ failure and chronic pain. Psychosocial disruption for the child, parents and siblings is frequent. Financial costs to the National Health Service are very high. The median waiting period for a heart is 193 days (95% CI 158–258), with younger children waiting the longest [1]. The significant limiting factor for transplantation is the shortage of organs and consequently, 25% of children will die whilst awaiting an organ [1]. Furthermore, in the current climate of organ scarcity, the more complex transplant candidates are denied access to listing as well as mechanical support due to negligible chance of ever being transplanted.

The clinical need exists not only in the realm of the recipient, but also in that of the donor. Organ donation brings a unique opportunity to find meaning in bereavement. Donations which are unable to proceed can bring disappointment to families [5, 6]. Many families gain comfort from knowing that their child’s death gave life to another child. Whilst most donor families do not meet their recipient, some do and report joy at hearing their child’s heartbeat again [7]. The heart, as is well recognised, has a special emotional significance for many.

## The History of Pediatric DCD Heart Transplantation

The first human heart transplanted by Christiaan Barnard in 1967, was from a DCD donor. After the establishment of brain-death criteria in 1968, virtually all donor hearts for the next 36 years were recovered from DBD donors until the beginning of the next millennium when DCD, or “non-heart beating donation” as it was known at the time, gained new interest.

In 2004, teams in Denver, Colorado performed three DCD infant heart transplants with 100% survival [8]. The circumstances surrounding the diagnosis of death ignited controversy and stimulated necessary robust debate on how donor death is determined [9].

It was subsequently shown, in large animal models, that even after the obligatory warm ischaemic insult during the standard DCD donation process, reperfusion of the retrieved *ex-situ* heart with oxygenated blood could provide transplantable organs [10].

In 2014, modern adult cardiac DCD transplantation commenced in Sydney with the use of direct recovery and reperfusion with oxygenated blood via *ex-situ* normothermic preservation utilising the TransMedics OCS™. The UK followed suit in 2015, led by the Papworth team and included a small number of older adolescents [11]. In 2019, clinical ethics panels from the two UK pediatric cardiac centres convened to discuss and approve cardiac DCD in children, and from 2020, children have been both cardiac DCD donors and recipients utilising the OCS (within the weight limitation of >50 kg) [12].

**FIGURE 4:**
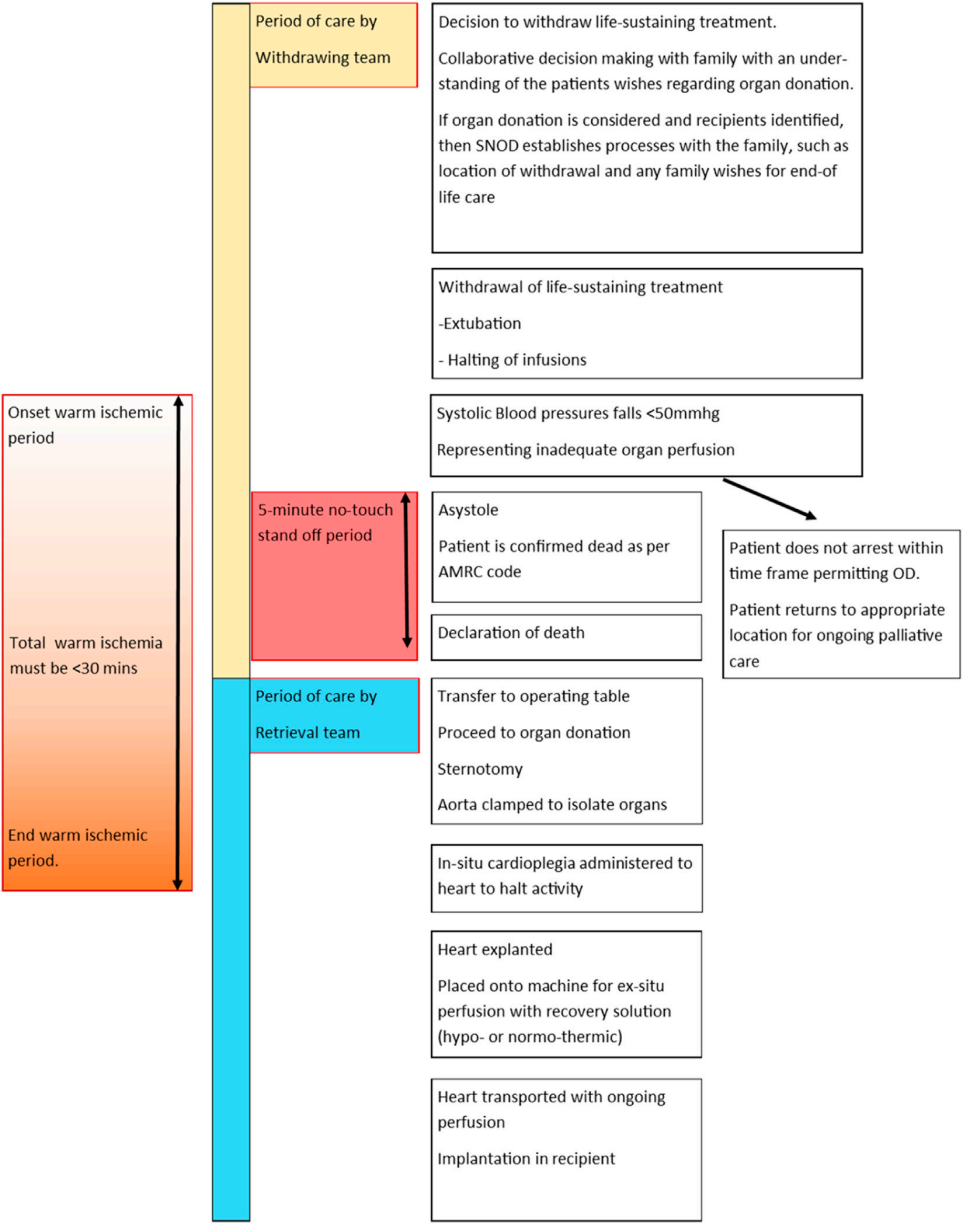
An infographic tracking the process of donation by circulatory death. In the UK a five-minute stand-off period is required following asystole. Recovery of the heart requires a functional warm ischemic time (from SBP<50 mmg or age-dependent pediatric equivalent) to onset of cardioplegia) to be less than 30 min.

The process of DCD organ recovery, including withdrawal of life-sustaining treatment (WLST), stand-off period and limitations on functional warm ischemia are identical for children as for adults and are clearly outlined in Figure 4.

In the past 5 years, teams across the globe have worked on advancing the technological options for supporting and expanding pediatric cardiac DCD donation [13–16]. There are now viable technologies to support the hearts of <50 kg donors with techniques of Normothermic Regional Perfusion *in situ* (NRP) and Hypothermic Oxygenated Perfusion *Ex-situ* (HOPE) having both been adopted internationally to permit cardiac DCD retrieval [13–22].

UK transplant centers seeking approval for these techniques have encountered previously resolved ethical concerns. These concerns, amidst other barriers which we seek to highlight in this paper, are preventing life-saving transplants from going ahead and need to be urgently resolved.

## What Are the Current Barriers to Cardiac DCD in Paediatrics in the UK?

It is widely acceptable, and medically feasible, for a child to receive a DCD donated heart, yet there are barriers when it comes to children becoming cardiac DCD donor. These barriers fall under three main categories: technological, resource and logistics, and ethical.

### Introduction of New Technologies

It is important to clarify that pediatric hearts are already being donated in the UK with the use of Direct Retrieval (DR) and normothermic *ex-situ* perfusion using the Transmedics OCS™. This technology is not able to perfuse hearts from donors <50 kg and consequently, due to permissible weight mismatching, for recipients >20 kg. The small-donor advancing field is focused on three alternative strategies: DR followed by *ex-situ* normothermic perfusion, DR followed by Hypothermic Organ Perfusion *Ex-situ* (HOPE), and *in situ* Thoraco-Abdominal Normothermic Regional Perfusion (TA-NRP).

#### Normothermic *Ex-Situ* Perfusion

The OCS™ is available for DCD heart recovery in donors >50 kg, with the main limiting factors being the aortic connector and concerns of perfusion pressure in smaller hearts. This system is utilised following DR for all DCD heart retrieval in the UK presently, including those of child donors >50 kg with excellent outcomes [4, 23, 24].

**FIGURE 5:**
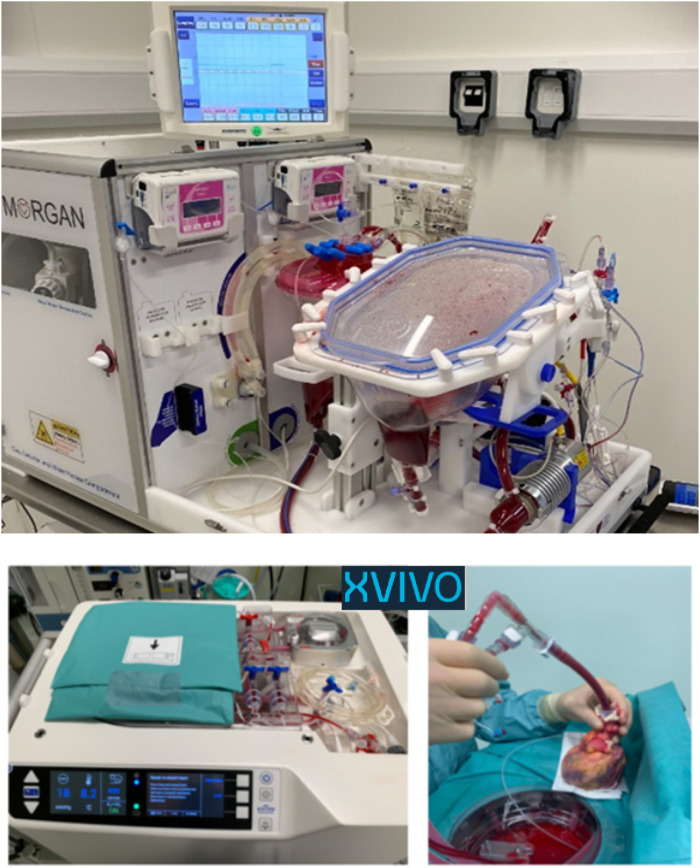
The two *ex-situ* perfusion technologies currently available to facilitate child DCD heart donation. The mOrgan utilizes normothermic continuous perfusion, and the XVIVO utilizes hypothermic continuous perfusion. Both devices are unlicensed and applications for their use have been under compassionate waiver.

In the drive to extend normothermic *ex-situ* perfusion to the child population, a collaboration between Royal Papworth Hospital and Great Ormond Street Hospital has resulted in “The mOrgan™” (Figure 5). This technology allows retrieval of any size heart down to a donor of 3 kg. Significant steps have been made toward operationalising the use of this device. Although experimental, this device was approved by regulatory bodies in March 2022 for a named patient on compassionate grounds. The named patient received 5 offers of hearts from pediatric DCD donors <40 kg, although none were suitable primarily due to logistics. Before a suitable DCD donor was identified, the child received a DBD donor heart. Despite clinical need and enthusiasm, the use of the mOrgan™ has not yet expanded beyond this case due to ongoing regulatory challenges, although a clinical trial is planned. To date, there are no published pre-clinical or clinical data for this device. Given the notable success of normothermic technology in the adult cardiac DCD programme, there is great enthusiasm for the potential the mOrgan offers to children.

#### Hypothermic Organ Perfusion *Ex-Situ* (HOPE)

Concurrently, the Newcastle team have been working toward utilising technology which permits the retrieved DCD heart to be re-perfused via Hypothermic Organ Perfusion Exsitu (HOPE) utilising the XVIVO Heart Assist Transport™ (Figure 5) [25, 26]. The XVIVO™ has been used on compassionate grounds for small child donors in the UK in both DBD and DCD pathways.

This approach uses small quantities of bank blood incorporated into a hyper-osmolar, potassium-rich hypothermic solution. It is thought that the avoidance of donor blood, together with low pressure allowed by the hypothermia avoids progressive myocardial oedema. Following cardiac DCD, continuous HOPE of the *ex-situ* donor heart is initiated.

Pre-clinical animal and human studies demonstrated restoration of metabolic performance and successful DCD heart transplantation with XVIVO™ [25, 27]. In the pre-clinical human studies, function of the DCD heart and biochemical normalisation of energy stores after reperfusion was comparable to the DBD heart [25]. Importantly, the animal studies compared DR + HOPE against NRP + HOPE, and NRP followed by cold static storage. The DR + HOPE had the best outcome, with better function than NRP followed by HOPE [27]. This may reflect the advantage of the initial perfusion being with hypothermic (8°C) blood and the avoidance of donor blood with associated cytokine and complement activation [28].

HOPE has been utilised to maintain prolonged perfusion, up to 12 h in DBD hearts with great success [29–31]. Additionally, the corresponding author reports using XVIVO for a small child DBD heart preservation (donor 15 kg) for 291 min perfusion with excellent clinical outcome following transplantation [26].

The Belgium group have published three cases of successful adult DCD heart transplant using XVIVO™ with excellent short-term outcomes [32]. In their ongoing programme, eight cases have been performed, including one adolescent case, with 100% 30-day survival (personal communication). Whilst the data for HOPE in DCD hearts appears promising, the early limitation was the size of the cannula. A collaboration between the Newcastle team and XVIVO led to development of a 14 mm cannula extending the opportunity to donate to small children and even infants.

Subsequently, in November 2024, humanitarian approval was given for a small child as the first UK DCD heart retrieval utilising direct procurement and XVIVO technology for recovery (lead clinician, corresponding author LK). Case reporting of this single case is pending, however early clinical outcomes are excellent with preserved ventricular function, no mechanical support requirement and full functional recovery of the child.

Since the XVIVO™ holds the heart in cold, static diastole with continuous low-pressure oxygenated perfusion, there is limited potential for ongoing assessment during perfusion. On the OCS™ or mOrgan™, the heart can be seen beating and serial lactate measurements can be performed. Whilst it is not possible to see the heart beating on XVIVO™, it is possible to measure lactate – the significance of which is debated. It is a poor predictor of cardiac function [33], particularly within a metabolically isolated organ [34]. There are similar questions regarding the validity of measuring function by eye-balling an unloaded beating heart.

More informative predictors of organ function are found in the donor medical history, the comorbidities, clinical status and mechanism of death. Total and warm ischemic time, the dying process and technical details are critical. The transplanting team must have confidence that a well-functioning heart exposed to a rigidly limited warm ischemic time and rapid retrieval process will be a good heart within the limitations of whichever *ex-situ* perfusion technology is used. The liver and kidney teams have taken this approach with excellent results – viewing donor management and organ preservation as a whole, rather than depending upon poorly validated techniques and biochemical markers [35–37].

Early evidence demonstrated by successful DCD recovery in UK and Belgium [32], in addition to small child DBD heart recovery [26] supports the hope that the XVIVO system is the solution for expanding paediatric DCD heart donation from children previously excluded, even down to organ recovery from neonates.

#### Thoraco-Abdominal Normothermic Regional Perfusion (TA-NRP)

In TA-NRP, an ECMO circuit is used to restore thoracic and abdominal oxygenated blood circulation within the donor body post-death) whilst isolating the brain from circulation [38]. The heart recommences beating and following a suitable period to allow metabolic recovery *in situ*, the heart can be assessed and retrieved using cardioplegia and cold-static-storage. Although TA-NRP, which permits perfusion and recovery of both abdominal and thoracic organs has been utilized in the UK historically, the thoracic component of TA-NRP was halted in 2020 due to ethical concerns and is subject to ongoing international debate for both adult and child donors [18, 38–41].

Abdominal-NRP (A-NRP), with the thorax isolated from the circulation, continues to be utilised in the UK to recover abdominal organs. Presently, in cases where A-NRP is adopted, the heart is recovered utilising DR and normothermic *ex-situ* perfusion with the OCS device.

TA-NRP-facilitated DCD heart transplant is practiced in Spain and the United States including neonatal donation [13–16]. Benefits for the organ and to the recipient are clear from the Spanish body of work which reports reduced warm ischaemic damage and superior assessment of organ viability [14, 15]. There are early reports of improved longer term survival following TA-NRP in comparison to DR-OCS although this is based upon small numbers [42]. Similar data is anticipated from centers in the USA which have adopted TA-NRP as the predominant method of cardiac DCD [43].

Reperfusion of the thoracic circulation, especially the restarting of the heart after death inside the body of the donor, raises controversy surrounding violation of the “dead donor rule.” There is additional concern over potential cerebral flow during recirculation resulting in the theoretical risk of restoring sentience in the donor. Inadvertent cerebral perfusion following death may result in an uncontrolled catecholamine storm with subsequent profound detrimental effect on all organs. Recent clinical research in human DCD donors has shown that perfusion pressure within the Circle of Willis does not increase upon initiation of TA-NRP with utilization of additional techniques to isolate the brain [44].

Nonetheless, these ethical concerns have led to a halt of TA-NRP in a number of European countries. A recent international consensus statement provides an excellent review of TA-NRP, the ethical dilemmas and the potential way forward [45]. In the UK, we await data from a validation study in Papworth and Cambridge University Hospital, regarding the prevention of cerebral perfusion, which will help inform ethical deliberation and professional consensus.

### Logistics and Resource Barriers

The DCD process depends upon a multifaceted, complex sequence: donor identification, referral to the Specialist Nurse in Organ Donation (SNOD), discussion with relatives, consent and often coronial approval, donor management for withdrawal of life-sustaining treatment, diagnosis of death, retrieval of organs, safe mounting of the organ onto the device, transfer, implantation and post-death care of the donor.

While much of the infrastructure required to support this process is well established at an individual hospital and national level, there are aspects of pediatric cardiac DCD which need attention.

#### Identification and Care of Donors

Reaching agreement with families to donate depends greatly on the attitudes and beliefs of healthcare staff. Where pediatric DCD has been adopted (UK, United States, Spain, Netherlands, Belgium, France) there is a positive attitude toward DCD donation across the disciplines and an understanding that donation contributes positively to a family’s grieving process [46–50]. Negative perceptions center around the complexity of the DCD process, poor knowledge of DCD protocols, perceiving withdrawal as professional failure, protection of children, fear that the donor feels pain and legal repercussions [46–54].

Child death and organ donation are highly sensitive, emotional topics. While the “lifesaving” act of donation can have a positive effect on grieving families, there is reported discomfort amongst healthcare providers in holding discussion regarding DCD which may impact upon donor referral and consent [46, 54].

The traditional approach to family-centred care and differences in end-of-life practice may conflict with what is needed for DCD [55]. DCD organ donation requires consideration of location and environment to minimise organ ischemic time. The concept of a witnessed, monitored death in an anaesthetic room adjacent to the operating theatre (the typical location for DCD in the UK), followed by an expedient move to theatre can be confronting to healthcare workers and donor families. These facts are discussed with the donor family as part of consent for donation and are justified by the guiding principle of “parental consent” and “overall benefit” when making decisions about end-of-life care [56, 57]. The family are always afforded the opportunity to be present and their privacy respected [58].

Decision-making is collaborative, with the healthcare team supporting the family. Whether a child has indicated willingness (e.g., by organ donor registration or through conversation) or has not expressed a view then pediatric clinicians are adept and accustomed to working collaboratively to reach a decision of best interests.

Understanding the reasons for families to decline DCD is helpful for recognizing how logistics and practicalities influence decision-making. In 2022-3, families of sixty-two dying children were approached regarding DCD organ donation. 44/62 (71%) of families were non-consenting. The most common reason was that parents wished to stay with their child after death or that their child had suffered enough. These reasons are also seen in DBD. A greater number approached for DCD felt the donation process to be too prolonged when compared to DBD [59].

Bespoke strategies are required to develop the environment and protocols to support staff in embracing pediatric DCD as part of end-of-life care [56]. In the UK, The Pediatric and Neonatal Deceased Donation Strategy embeds organ donation as a routine end-of-life choice for every family facing the death of their child [60]. The multidisciplinary leadership course “Child and Infant Deceased Donation” trains clinical teams to confidently use the national strategy recommendations within their practice, to transform cultures and develop policy through local leadership.

The impact of such robust national recommendations is illustrated in all-age DCD donation statistics in the UK which followed government strategies in 2008 and 2013 to increase deceased donation [61, 62]. The number of families approached from 2007 to 2012 increased by 4% for DBD (1,055 to 1,100) but increased by 420% for DCD (349 to 1,816), resulting in a 154% increase in the number of DCD donors (200 to 507) over the same 5 years [1].

Since 2010, more families in the UK consent to DCD each year than to DBD [1]. This increase is a direct result of a cultural shift in ICU attitude and behaviours toward DCD, empowered by nationally endorsed strategic planning and recommendations [59, 61–63]. Staff involved need to be educated about the process and confident of the legal framework for DCD pediatric organ donation provided primarily by the Human Tissue Act 2004 and follow-up guidance [56, 64, 65].

#### Infrastructure and Resources for Organ Recovery

The UK National Organ Retrieval Service (NORS) was established by NHSBT in 2010 to provide a 24-h national service for deceased donation. Two specialist pediatric teams in the UK retrieve hearts from DBD donors <40 kg. The established DCD programme only retrieves hearts from donors, including children, over 50 kg. Currently, only one of the specialist pediatric retrieval teams has the additional expertise to retrieve DCD hearts. A formally commissioned, national DCD heart programme is awaited. Until the DCD heart retrieval service is sustainably funded and formally commissioned, there is a financial and logistical barrier to new technologies.

Cardiac DCD retrieval is a resource-intensive endeavour requiring theatre space, personnel, devices and disposables and often private air-travel. However, it must be weighed against the cost of mechanically supporting a child on the heart transplant waiting list. The cost of a Ventricular Assist Device (VAD) supported pediatric journey to heart transplant is upwards of US$700,000 [66]. Investment in processes to increase the number of donor hearts available and improve organ utilisation rates is in itself, a viable financial argument.

In 2018, a commitment was made to ensure consistently available expertise and skill to retrieve organs from all pediatric patients including small infants. While this did not specify DCD, the recommendations do state that ongoing clinical governance processes should review specific challenges, and ongoing training needs to achieve this commitment [59]. In 2023, as work toward viable technology and infrastructure progressed, the UK National DCD Pediatric Working Group was convened to establish the logistical barriers to cardiac DCD in children, including the necessary collaboration and training required to establish a complete retrieval team.

It is inevitable that both pediatric heart recovery teams will need to be DCD trained in order to sustain a safe cardiac DCD programme for children, however, the limitation of national sustainable funding for the DCD heart service is impeding the progression of any pediatric DCD heart programme.

### Ethical Barriers

There is considerable variability in ethical perspectives on DCD organ donation across the globe [19, 40, 41, 67–76]. Focusing on countries with an established deceased donation programme, those who question DCD heart donation raise concerns related primarily to the diagnosis of death, the permissibility of restarting the heart and whether DCD, particularly TA-NRP, involves breaching of the dead donor rule [77–79]. The acceptance of the ethics of DCD heart donation in adult practice within the UK is demonstrated by the breadth of professional, legal and ethical documents available from The Department of Health, Royal Colleges, the General Medical Council, the National Institute for Health and Care Excellence (NICE), the UK Donation Ethics Committee (UKDEC), the Intensive Care Society, NHS Blood and Transplant (NHSBT) and the British Transplant society [9, 45, 56–58, 62, 65, 80–89].

Progress on the technological front to facilitate pediatric DCD has led to situations in the UK where previously settled ethical concerns have been questioned again. Although notably the questions raised have been no different when it comes to children, it is only that there is a new audience confronting the ethics for the first time. Given the acceptance by the medical community and society for adult cardiac DCD, it could be considered unethical and even discriminatory to deny the opportunity for transplant in children based upon the same ethical principles. As stated by NICE, the GMC, The Royal College of Pediatrics, NHSBT, the Pediatric Intensive Care Society, and UKDEC, organ donation should be a routine component of a child’s end of life care and as such it should be considered in any child in whom the decision has been made for withdrawal of life support [56, 65, 83, 86–89].

In 2015 UKDEC published a position paper on ethical issues in pediatric organ donation [56]. Nine recommendations reinforce the importance of facilitating donation where a family wishes to. The positives of child organ donation are well documented. For many, the single positive outcome of their tragedy is their child’s potential to save others [59]. Empowering families to explore their feelings and take control of decisions around donation can have a significant effect on meaning-making and healing [90].

Ethical dilemmas in DCD lie in the grounds of potential conflict between what is right for the individual as a dying patient, what is right for the individual as an organ donor and for the family who are giving their consent. With the widespread ethical, legal and professional support, resulting in nearly 10,000 DCD donations in the UK over the last 24 years [1], we must acknowledge that though new technology can raise new questions, the fundamental questions have been met with robust and reflective ethical answers and this is a practice widely accepted by families and clinicians in the UK and our international peer nations.

## Summary and a Call to Action

The emergence of technology dedicated to the *ex-situ* perfusion of small hearts has been long-awaited and now requires prioritisation in order that children can have the same opportunity for a life-saving transplant as adults. HOPE has now been utilised in the UK for a small child DCD heart donation and transplant with excellent result and as such it is time to address all barriers to ensure equitable access for children. We can no longer deny DCD hearts to children on the basis of lack of technology.

Logistical barriers of donor identification and care, organ retrieval and resources can be overcome. There is however an urgent need to communicate the message to decision-makers about cardiac DCD technology, that the fundamental ethics of DCD are already well established. Cardiac DCD is embedded practice in adults in the UK and there is no rational argument for difference in pediatric practice. Indeed, it would seem to be unethical to withhold life-saving technology from children who need it. A 25% mortality on the transplant waiting list is unacceptable when a solution exists, and which would be available to children if they were just a few years older. We have a responsibility to children and families, who are donating other organs using DCD processes, to allow them to donate the heart too.

We call upon the Department of Health, Royal College of Paediatrics and Child health, The British Transplant Society, NHSBT, and international equivalents to demand urgent action to:

• Ensure that no child dies unnecessarily due to failure to provide appropriate services analogous to those available to adults and older children.
• Build the logistical framework to facilitate pediatric cardiac DCD within the already established ethical, legal and professional frameworks.
• Provide education and training of all staff involved in this complex process.
• Ensure a sustainable organ retrieval service in order that no organ is lost due to skill deficit by training both pediatric retrieval centers to undertake cardiac DCD.
• Apply the new technologies under appropriate surveillance, safety monitoring and rigorous reporting to the clinical community across both paediatric heart transplant centres in the UK.
• Urge NHS commissioners to recognise the financial benefit of employing technology to increase the donor pool for young children on the waiting list and seek sustainable funding for DCD paediatric heart recovery.
• Demand due process from the regulatory health authority to allow for compassionate use of technology to prevent further loss of life due to delay.
